# PPARα Signaling: A Candidate Target in Psychiatric Disorder Management

**DOI:** 10.3390/biom12050723

**Published:** 2022-05-20

**Authors:** Simona Scheggi, Graziano Pinna, Giulia Braccagni, Maria Graziella De Montis, Carla Gambarana

**Affiliations:** 1 Department of Molecular and Developmental Medicine, University of Siena, Via Aldo Moro 2, 53100 Siena, Italy; giulia.braccagni@student.unisi.it (G.B.); mariagraziella.demontis@unisi.it (M.G.D.M.); carla.gambarana@unisi.it (C.G.); 2 Department of Psychiatry, The Psychiatric Institute, University of Illinois Chicago, Chicago, IL 60607, USA; gpinna@uic.edu

**Keywords:** N-acylethanolamines, endocannabinoids, neurosteroids, fenofibrate, dopamine, neuroinflammation, major depression, anhedonia, schizophrenia, autism

## Abstract

Peroxisome proliferator-activator receptors (PPARs) regulate lipid and glucose metabolism, control inflammatory processes, and modulate several brain functions. Three PPAR isoforms have been identified, PPARα, PPARβ/δ, and PPARγ, which are expressed in different tissues and cell types. Hereinafter, we focus on PPARα involvement in the pathophysiology of neuropsychiatric and neurodegenerative disorders, which is underscored by PPARα localization in neuronal circuits involved in emotion modulation and stress response, and its role in neurodevelopment and neuroinflammation. A multiplicity of downstream pathways modulated by PPARα activation, including glutamatergic neurotransmission, upregulation of brain-derived neurotrophic factor, and neurosteroidogenic effects, encompass mechanisms underlying behavioral regulation. Modulation of dopamine neuronal firing in the ventral tegmental area likely contributes to PPARα effects in depression, anhedonia, and autism spectrum disorder (ASD). Based on robust preclinical evidence and the initial results of clinical studies, future clinical trials should assess the efficacy of PPARα agonists in the treatment of mood and neurodevelopmental disorders, such as depression, schizophrenia, and ASD.

## 1. Introduction

Peroxisome proliferator-activator receptors (PPARs) belong to the type II nuclear hormone receptor superfamily and primarily regulate the transcription of genes involved in cellular metabolism and energy homeostasis, as well as genes involved in inflammatory processes and oxidative stress [1]. They are also abundantly expressed in the central nervous system (CNS), where they are involved in the regulation of several neuronal functions, including modulation of affective behavior [2]. Three isoforms have been identified within the PPAR family, including PPARα, PPARβ/δ, and PPARγ, that share a high degree of structural and sequence homology [3] and are encoded by separate genes (PPARα, NR1C1, PPARβ/δ, NR1C2, and PPARγ, NR1C3) located on chromosomes 15, 17, and 6 in the mouse and on chromosome 22, 6, and 3 in humans, respectively [4,5]. The name of these receptors derives from the initial observation that their activation induces hepatic peroxisome proliferation in rodents. However, this effect is limited to PPARα, the first isoform to be identified, which is not observed in humans [6]. The three isoforms expressed in rodents share 92–98% homology with the respective human receptor [4].

**Figure 1:**
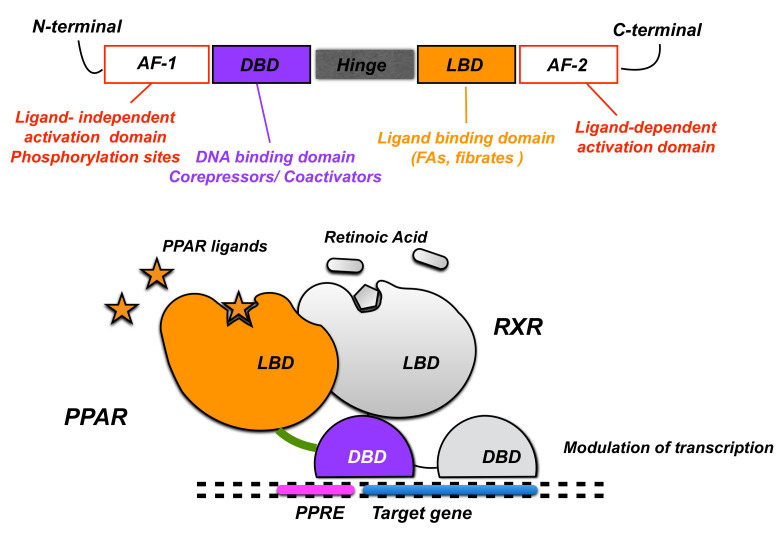
Gene structure of PPAR and regulation of transcription.

Upon interacting with their specific ligands and following their activation, PPARs form heterodimers with the 9-cis retinoic acid receptor (RXR) [7] and modulate transcription by binding to the putative specific peroxisome proliferator response elements (PPREs) in the promoter regions of target genes [8]. PPARs share the same protein structure that contains different functional domains. The N-terminal domain that harbors a ligand-independent activation function 1 (AF-1) is responsible for the phosphorylation of the receptor (Figure 1). The DNA-binding domain consents PPAR binding to PPREs in the promoter region of genes. The hinge domain mediates intracellular trafficking, and the C-terminal ligand-binding domain is responsible for the ligand specificity and activation of PPAR and also contains the ligand-dependent activation function 2 (AF-2) that allows the recruitment of PPAR co-activators and co-repressors to modulate gene transcription [9]. The stability and transcriptional activity of PPARs is regulated by processes of phosphorylation, ubiquitylation, and SUMOylation [10].

PPARs show a wide distribution in different tissues and cell types, with partial overlap between the several isoforms. PPARα is highly expressed in the liver, heart, and brown adipose tissue, where it regulates the fatty acid oxidation pathways, ketogenesis, and lipid storage. PPARβ/δ has a ubiquitary expression and plays a crucial role in fatty acid and glucose oxidation, in particular in the liver, skeletal muscle, and heart. PPARγ shows high expression levels in the white adipose tissue, where it regulates adipogenesis, and is a potent modulator of whole-body lipid metabolism and insulin sensitivity [11]. Important regulatory roles have also been ascribed to PPARs in cell proliferation and differentiation, development, inflammatory processes, and tumorigenesis [12,13,14,15].

The main endogenous ligands of PPARs are polyunsaturated fatty acids and several lipid molecules, such as saturated fatty acids, eicosanoids, leukotrienes, oxidized fatty acids, and oxidized phospholipids, are all regarded as PPAR activators [16,17]. Ligands including the fatty acid ethanolamides, or N-acylethanolamines (NAEs), such as the endocannabinoid arachidonoylethanolamide, or anandamide (AEA), palmitoylethanolamide (PEA) and oleoylethanolamide (OEA), regulate anti-inflammatory and antinociceptive functions and feeding behavior [18]. While AEA can bind to PPAR and activate its transcriptional activity, it shows only a modest affinity for PPARα [19,20]. On the other hand, PEA and OEA show affinity for PPARα in the nanomolar range [21]. Technically, PEA is considered an endocannabinoid-like modulator in that, unlike AEA, it fails to activate classical cannabinoid receptor (CB), including CB1 and CB2. However, PEA can influence the activity of AEA at CB1, and PPARα itself is considered an endocannabinoid target. PEA also shows agonistic properties by binding at GPR55, which plays a role in the anti-inflammatory and behavioral effects of endocannabinoids [22].

Among synthetic PPARα ligands, the fibrates, such as fenofibrate, are used in the treatment of dyslipidemia, and PPARγ ligands (glitazones) are used as antidiabetic drugs, while PPARβ/δ ligands do not yet have clinical indications [23]. More recently, dual PPAR α/γ, α/δ, and γ/δ agonists have been developed in the effort to combine the therapeutically useful effects and mitigate the drawbacks of isotype-specific agonists in the treatment of complex diseases. PPARα/γ dual agonists (e.g., glitazar) and PPARα/δ dual agonists (e.g., elafibranor) have been developed for clinical use in adults with primary biliary cholangitis, and pan-PPAR agonists are under Phase III clinical trials for nonalcoholic steatosis (e.g., lanifibranor, a PPARα/γ/δ agonist) [23].

This review focuses on the emerging role played by PPARα in neuropsychiatric and neurodevelopmental disorders, including depression, schizophrenia, and autism spectrum disorder (ASD), and in neurodegenerative diseases, such as Alzheimer’s and Parkinson’s disease. The role of PPARα in the CNS encompasses the regulation of neural cell differentiation, neurogenesis, neuronal development, and neuroinflammation [24], as well as modulation of several complex physiological functions, such as memory consolidation and pain perception [25]. Thus, these nuclear receptors have been considered as potential targets to treat neurodegenerative and neuropsychiatric disorders, including drug-related behaviors [26,27,28,29].

## 2. PPARα Expression and Physiological Role in the CNS

PPARα is expressed in the rodent brain from embryonic stages of development to adult life [30]. In the adult rodent and human brain, PPARα is the only isotype that can be detected in all three cell types (neurons, astrocytes, and microglia), although its most prominent expression is in neurons, where it mainly shows a nuclear localization [31]. In glial cells, PPARα shows a higher expression in astrocytes, where it is localized in the cell body and astrocytic processes, than in microglia [31]. Moreover, PPARα has a discrete regional distribution, with the highest levels of expression in the prefrontal cortex (PFC), basal ganglia, nucleus accumbens (NAc), amygdala, ventral tegmental area (VTA), and thalamic nuclei; lower expression levels are detected in the hippocampus [31,32]. In the hippocampus, PPARα has been localized in the CA1, CA2, and CA3 and in dentate gyrus regions [28]. The discrete pattern of expression is consistent with a possible role played by PPARα in the modulation of the ventral striatal and extended amygdala circuits that are involved in reward and motivational responses, and thus in mood disorders and anhedonia [31].

Although PPARα null mice develop normally and do not show apparent behavioral abnormalities in standard experimental conditions [33], PPARα plays important physiological roles in the CNS, consistent with its expression in microvasculature, in neuronal and glial cells. This receptor and its endogenous ligands, e.g., OEA and PEA, are involved in the regulation of energy homeostasis and modulation of neuroinflammation, glial cell proliferation/differentiation, antioxidant responses, and neurogenesis, and they even affect neurotransmission [28].

Thus, the pleiotropic physiological effects of PPARα activity in the CNS suggest a potential protective role of PPARα agonists in neuropsychiatric disorders and neurodegenerative diseases, and this system has become a relevant topic for basic and clinical research as a novel therapeutic strategy for the treatment of diverse CNS pathological conditions.

### 2.1. Modulation of Neuroinflammation

Anti-inflammatory effects mediated by PPARα in the CNS involve the transrepression of pro-inflammatory transcription factors such as nuclear factor kappa-light-chain-enhancer of activated B cells (NFkB), that results in the inhibition of inflammatory cytokine release, such as tumor necrosis factor-α (TNF-α) and interleukins 1 and 6. In the microvasculature, PPARαs reduce the expression of cyclooxygenase-2 and cytokine-induced expression of vascular cell adhesion protein 1 (VACAM-1) and promote the expression of endothelial nitric oxide synthase (eNOS) [25,34].

### 2.2. Neuroprotection

The neuroprotective effects observed following PPARα activation may relate to the modulation of glutamatergic transmission. Through transcriptional regulation of cyclic AMP response element binding (CREB), PPARα controls the oscillations of calcium influx and the expression of genes coding for *N*-methyl-d-aspartate (NMDA) receptor subunit NR2A, NR2B, and the 2-amino-3-(3-hydroxy-5-methyl-isoxazol-4-yl) propionic acid (AMPA)-receptor subunit GluR1 in mouse hippocampal subregions (CA1, CA2, CA3, and dentate gyrus, DG) [35]. Furthermore, there is evidence that PPARα ligands increase the internalization of the glutamate transporter GLT-1 in astrocytes, modulating the mechanisms subserving astrocyte-mediated glutamate homeostasis in the brain [36]. In addition, the neuroprotective effects elicited by PPARα may also be mediated by increasing levels of brain-derived neurotrophic factor (BDNF) reported after the administration with PPARα agonists [37]. New endogenous PPARα ligands have been isolated in hippocampal neurons [38], and one of these ligands, hexadecanamide, upregulates BDNF expression in hippocampal neurons, thus promoting synaptic functions and plasticity, as reported in a mouse model of Alzheimer’s disease (AD), the 5XFAD mouse [39].

### 2.3. Regulation of Neurotransmission

PPARα participates in the regulation of complex CNS responses, such as satiety, spatial learning, and long-term memory, and modulation of nociception [25]. The positive modulatory effects of PPARα activity on memory at the hippocampal level have been related to the regulation of gene expression of cyclic AMP response element binding (CREB) and CREB-dependent plasticity-related molecules, such as the NMDA receptor subunit NR2A [35].

Moreover, both acute and repeated stimulation of PPARα controls β2-subunit-containing nicotinic receptors (β2nAChRs) in dopaminergic neurons in the VTA, thus affecting dopaminergic activity in terminal regions [40,41]. The mechanisms activated by PPARα stimulation have also been implicated in the orchestration and modulation of stress responses [42], and these effects are at least in part due to the enhanced biosynthesis of neurosteroids [43]. The observed correlation between PPARα activation and the biosynthesis of neurosteroids, such as allopregnanolone/pregnanolone, suggests a role for PPARα and its downstream effectors in the modulation of complex responses to stressful events and led to the proposal of this system as a possible target for the treatment of the severe emotional dysfunctions that characterize post-traumatic stress disorder (PTSD) and major depression [42,43].

## 3. PPARα in CNS Disorders

The localization of PPARα in neuronal circuits involved in modulating emotions and responses to stress [32,44] suggests that PPARα may play a role in the pathophysiology of neuropsychiatric and neurodegenerative disorders whose ethiopathogenesis involves cell metabolic alterations, oxidative damage, and neuroinflammation (summarized in Table 1 and Table 2) [27,43,45,46,47,48,49,50,51,52].

### 3.1. Role of PPARα in Depression and Anhedonia

Major depression is a condition characterized by negative mood states with an increase in negative affect and decrease in positive affect. Anhedonia has emerged as an appropriate endophenotype of depression [53,54] and is considered a predictor of increased risk of death and disability in major depression patients. Indeed, anhedonia is linked to worse treatment outcomes for these patients [55,56,57].

Stimulating PPARα signaling induces potent behavioral effects and may offer a suitable treatment strategy to improve both symptoms of depression and anhedonia in patients. In preclinical studies, antidepressant effects have been observed after administration with several PPARα agonists, for instance, after administration with the PPARα endogenous modulator, PEA. For instance, both fluoxetine and PEA given for a week to rodents yielded comparable antidepressant effects [58]. In the corticosterone model of depression, PEA administered for 14 days resulted in strong antidepressant effects. Administered over two weeks, PEA also induced antidepressant effects in a neuropathic pain rodent model and in a mild traumatic brain injury (mTBI) rat model aggravated by depression-like behaviors [59]. Synthetic PPARα ligands, such as WY-14643, decrease neuroinflammation and oxidative stress, and elicit antidepressant effects in a model of lipopolysaccharide (LPS)-induced depressive-like behaviors [60]. WY-14643 blocked production of cytokines, prevented the LPS-induced oxidative and nitrosative stress, and blocked the LPS-induced decrease in BDNF in the hippocampus and prefrontal cortex [60].

The investigation of PEA antidepressant effects has since been evaluated in a clinical study that used ultramicronized PEA given to patients for 6 weeks in a placebo-controlled clinical trial as an add-on treatment with citalopram [61] (Table 2). This treatment resulted in a significant antidepressant effect after a 2-week treatment and the efficacy of PEA persisted until week 6 of treatment. Subjects who received PEA reported fast antidepressant effects associated with a high response rate and negligible side effects. While these results are intriguing, large-scale clinical trials should replicate these findings.

Herein, we examine several mechanisms by which targeting PPARα regulates the pathophysiology of depression and anhedonia.

#### 3.1.1. PPARα Regulation of VTA Dopamine Neurons

A primary mechanism by which PPARα may be involved in the regulation of major depression and anhedonia consists of the regulation of VTA dopamine neuronal firing. Anhedonia has been associated with blunted dopaminergic transmission to pleasurable stimuli and reduced reward responsiveness. Particularly, the activation of dopaminergic mesolimbic pathways, originating in the VTA and projecting to the NAc, which assigns incentive values to reward-associated cues, has been consistently considered crucial for reward processing and motivated behaviors [62,63,64,65]. Indeed, clinical imaging studies have demonstrated a reduced NAc activation in depressed patients in response to rewarding stimuli [66,67] and weaker functional connectivity with other regions involved in mood regulation [68,69]. On the same line, preclinical studies show that in rats a condition of stress-induced motivational anhedonia, assessed by sucrose self-administration, is accompanied by blunted dopaminergic responses to sucrose reward in the shell of the NAc [70]. Moreover, reinstatement of reward motivation by several pharmacological treatments (lithium, imipramine, clozapine, aripiprazole, lamotrigine, and fenofibrate) is associated with restored dopaminergic response to sucrose in the NAc shell, assessed by increased phosphorylation of dopamine and cAMP-regulated phosphoprotein, Mr 32,000 (DARPP-32) [70,71,72,73].

Several inputs are involved in the modulation of dopamine neurons in the mesocorticolimbic circuit: (i) the VTA GABAergic neurons, which provide an inhibitory innervation to dopamine neurons [74], (ii) the glutamatergic afferents to VTA and the presence of local glutamatergic neurons [75], which promote positive reinforcement by increasing dopamine release in the NAc, (although recent data suggest that glutamate release in the VTA is *per se* sufficient to promote positive reinforcement with a mechanism independent from dopamine release [76]), and (iii) the nicotinic cholinergic transmission. Particularly, the latter seems to play a prominent role in the modulation of VTA dopaminergic neuron activity, since nAChRs are abundantly expressed in this area and are strategically located on glutamatergic and dopaminergic VTA neurons, as well as on pre-synaptic terminals from other afferent pathways to VTA and on GABAergic interneurons [77,78]. nAChRs are also present on dopaminergic terminals in the NAc and mPFC [77,79]. The β2nAChR plays a critical role in controlling the firing rate of dopaminergic neurons in the VTA [80], and PPARα activation modulates the activity of β2nAChRs expressed in VTA dopaminergic neurons. Thus, PPARα serves as an intrinsic regulator of cholinergic transmission, indirectly controlling the firing of midbrain dopamine neurons. Moreover, a cross talk between PPARα and the β2nAChRs in VTA dopaminergic neurons has been demonstrated by electrophysiological studies in rats that showed that acute PPARα stimulation reduces the firing of VTA dopamine neurons by increasing the phosphorylation levels of the β2 subunit of nAChRs. This effect is absent in β2 subunit knockout mice and is restored when β2nAChRs are selectively re-expressed in VTA dopamine neurons [40,41].

**Figure 2:**
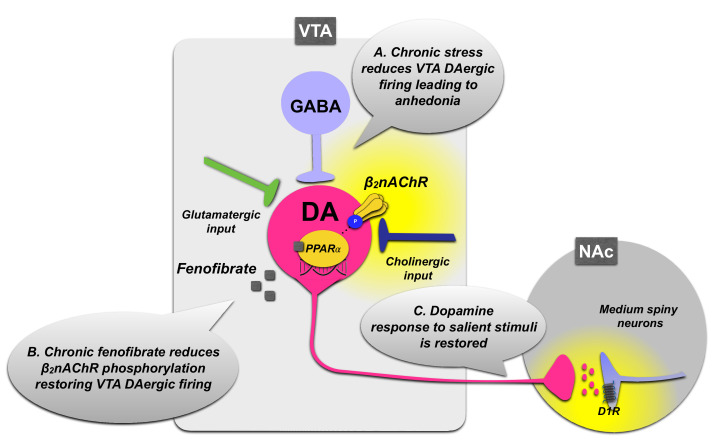
Schematic representation of PPARα regulation of dopamine neuron activity in the ventral tegmental area (VTA). (**A**) Unavoidable stress exposure is associated with decreased burst firing of dopamine neurons in the VTA and impaired dopaminergic response in the NAc. (**B**) Long-term PPARα activation by agonist administration (fenofibrate) decreases phosphorylation levels of β2-subunit-containing nicotinic acetylcholine receptor (β2nAChR) in the VTA, thus counteracting the negative modulatory effect on dopaminergic transmission of phosphorylated β2nAChRs. The decreased phosphorylation levels of β2nAChRs lead to increases in burst firing of VTA dopamine neurons (**C**), an effect that is accompanied by increased dopaminergic responses to salient stimuli in the nucleus accumbens (NAc). PPARα activation by relieving the inhibition of VTA dopamine neurons may restore the NAc physiological response to salient stimuli.

On these grounds, it has been proposed that PPARα activation may elicit antidepressant-like effects. In order to test this hypothesis, the modulatory effect of PPARα on VTA dopamine neuron firing has been examined in rats following repeated receptor activation. Long-term activation of PPARα by the synthetic agonist fenofibrate induces a decrease in the phosphorylation levels of β2nAChRs that is accompanied by phasic activation of spontaneously active dopamine neurons [72]. Moreover, the switch in the firing patterns of VTA dopamine neurons elicited by long-term fenofibrate administration is accompanied by the relief of the depressive phenotype induced by unavoidable stress exposure and the reinstatement of the dopaminergic response to salient stimuli in the NAc [72]. Indeed, chronic stress protocols based on rodent exposure to unavoidable nociceptive stimuli have been associated with depression of VTA dopamine neuron activity and impaired burst firing that would affect the dopaminergic response to salient stimuli in the NAc [81,82]. These modifications in dopaminergic transmission are considered as correlates of the behavioral depressive-like phenotypes of hyporeactivity and anhedonia. Long-term stimulation of PPARα induced by fenofibrate administration may decrease the negative modulatory action on β2nAChRs, likely due to a mechanism of receptor desensitization, thus relieving VTA dopamine neurons from inhibition (Figure 2). In turn, this may restore the physiological activity and burst firing of VTA neurons in response to stimuli and increase dopamine release at target projection regions, such as the NAc, at high enough levels to stimulate D1 dopamine receptor transmission. The increase in D1-dopamine-receptor-dependent signaling, and in particular in PKA-dependent phosphorylation of the Thr34 residue of DARPP-32, is observed in the NAc in response to positive salient stimuli, and its restoration accompanies the reinstatement of motivated behavior [72,83,84,85] (Figure 2). Consistent with this hypothesis, optogenetic stimulation of NAc medium spiny neurons expressing D1 dopamine receptors resulted in stress resilience [86]. Therefore, these observations suggest that the repeated administration of PPARα agonists may exert antidepressant-like effects by restoring the VTA-NAc dopaminergic circuit, whose activity is instrumental to reinforcement learning. In line with this role of PPARα in the modulation of dopaminergic transmission, PPARα knockout mice displayed enhanced fear learning through a D1-dopamine-receptor-dependent mechanism, and increased levels of extraneuronal dopamine in the amygdala [87].

#### 3.1.2. Role of PPARα Expression in the Hippocampus

A second hypothesis regarding dysfunctional neurocircuitry, and possible mechanisms that may contribute to the pathophysiology of depression, points to the role of hippocampal PPARα expression. Indeed, exposure to different chronic stress protocols that induce a depressive-like phenotype in rodents, including social defeat, unavoidable stress, and social isolation, all significantly decreased PPARα expression in the hippocampus, but not in other regions, such as the mPFC, NAc, VTA, and amygdala [88,89]. For instance, a stress-induced epigenetic PPARα downregulation was recently reported with hypermethylation of its promoter region [51]. In support of this hypothesis, when hippocampal PPARα expression is downregulated in the mouse by an shRNA infusion, immobility in the forced swim test is enhanced, which is predictive of a pro-depressant activity [88]. Conversely, in mice exposed to social defeat, AAV-mediated hippocampal PPARα overexpression decreased the immobility time, which is predictive of an antidepressant effect. This intervention also restored sucrose preference and social interaction to control levels [88]. 

**Figure 3:**
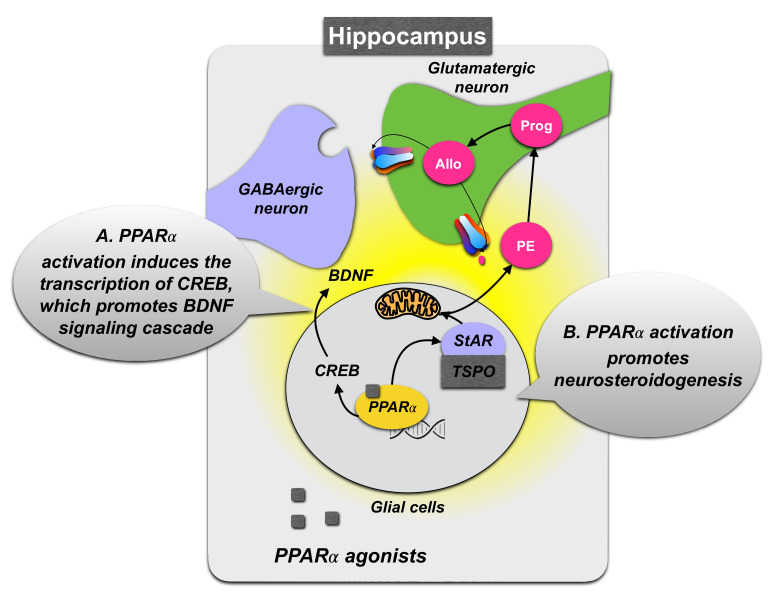
Schematic representation of PPARα signaling pathway in the hippocampus following agonist administration. (**A**) PPARα activation, upon dimerization with the retinoid receptor (RXR), induces transcriptional regulation of cyclic AMP response element-binding protein (CREB), which promotes the stimulation of brain-derived neurotrophic factor (BDNF) signaling pathway, resulting in antidepressant-like effects and improving learning and memory. (**B**) PPARα activation can also induce allopregnanolone (Allo) biosynthesis by upregulating neurosteroidogenic enzymes and proteins, including the steroidogenic acute regulatory protein (StAR). The interaction of StAR with cholesterol and translocator protein (TSPO) leads to cholesterol entry into the mitochondria and its conversion into pregnenolone (PE). In hippocampal glutamatergic pyramidal neurons, PE is transformed into progesterone (Prog) and then Allo, which, by interacting with GABA_A_ receptors, modulates emotional behavior.

A mechanism underlying PPARα function in the expression of depression-like phenotypes may depend on BDNF expression. Activation of PPARα was reported to enhance the transcriptional activity of CREB and promote CREB-mediated biosynthesis of BDNF and other neuroplasticity-related proteins [88] (Figure 3). Accordingly, in experimental models, a depressive-like phenotype is often accompanied by reduced BDNF levels in the hippocampus [90], and the chronic administration of PPARα synthetic agonists (fenofibrate and gemfibrozil) or the endogenous agonist PEA can revert stress-induced behavioral modifications and normalize hippocampal BDNF signaling decreased by stress exposure [91,92,93]. Furthermore, the PPARα-CREB-BDNF pathway may have a crucial role in the mechanisms underlying the positive effects of antidepressant treatment. Long-term fluoxetine, venlafaxine, and vortioxetine treatment upregulates PPARα expression levels in the hippocampus in mice exposed to chronic stress models [37,88,89], suggesting that these antidepressants modulate hippocampal PPARα function by mechanisms including activation of serotonin/noradrenaline-mediated pathways. However, they may also directly bind to and activate PPARα [37,88,94].

#### 3.1.3. PPARα Neurosteroidogenic Effects

The antidepressant effects elicited by PPARα activation may also encompass other signaling pathways, including those mediated by neurosteroids and endocannabinoids. Indeed, in vitro studies showed that PPARα stimulation by PEA induced the upregulation of neurosteroid biosynthesis [95] (Figure 3). PEA action appeared to be mediated by enhancing the expression of crucial neurosteroidogenic enzymes, leading to an increase in peripheral allopregnanolone concentrations, quantified in the spinal cord [96]. This effect normalized pentobarbital-induced sedation and was blunted by PPARα selective antagonists and allopregnanolone biosynthetic enzyme blockers, such as administration with the 5α-reductase inhibitor, finasteride. Consistent with these findings and banking on the widespread expression of PPARα in several corticolimbic areas deputed to the regulation of affective-like behavior, other studies have further evidenced the behavioral effects of PPARα activation through the capacity of this target to upregulate neurosteroidogenesis. In an animal model of protracted stress-induced depression and PTSD, the socially isolated mouse, a single dose of PEA upregulated levels of allopregnanolone in several corticolimbic areas, including the frontal cortex, amygdala, hippocampus, and also in the olfactory bulbs. This treatment efficiently attenuated aggressive behavior as well as depressive-like and anxious-like behaviors [43,48]. Furthermore, in a contextual fear conditioning paradigm, PEA, administered immediately after a fear reactivation session, facilitated fear extinction and fear extinction retention by a mechanism including a reconsolidation blockade. These results were supported by behavioral pharmacology experiments showing that PEA anxiolytic and antidepressant effects could be mimicked by administration with the synthetic PPARα selective agonist, GW7647 or fenofibrate. Furthermore, the behavioral effects following PEA administration could be blunted by pre-administering finasteride that also blocked the PEA-induced allopregnanolone upregulation in the hippocampus, and by a genetic excision of PPARα [43].

### 3.2. Role of PPARα in Autism Spectrum Disorder (ASD)

ASD encompasses a heterogeneous group of neurodevelopmental disorders with multiple genetic and environmental etiologies, characterized by persistent deficits in social communication and interaction, and repetitive patterns of behavior, with restricted interests or activities [97]. Despite the heterogeneity of the phenotypes and complexity of the pathophysiology of ASD, some of the mechanisms underlying the development and progression of this disorder have been suggested. In particular, the possible role of altered inflammatory responses and glutamatergic transmission is supported by several clinical observations [98,99,100]. The hypothesis that neuroinflammation contributes to the pathogenesis of ASD suggested investigations into the potential role of PPARs, and in particular of PPARα, in the pathophysiology of ASD. The decreased expression and activity of PPARα may result in excessive production of proinflammatory cytokines and decreased expression of antioxidants, resulting in increased oxidative stress that eventually disrupts the cellular integrity and alters neuronal function. 

This role of PPARα is now supported by a number of studies on different animal models of ASD, demonstrating that the presence of behavioral deficits, reminiscent of autistic core symptoms, are accompanied by reduced PPARα expression levels in several brain regions, including the PFC and hippocampus [101,102,103]. Reductions in PPARα levels are often associated with an increase in the expression of markers of neuroinflammation and oxidative stress, and glial cell reactivity consistent with the opposite effects of prevention or reduction in oxidative damage and neuroinflammation elicited by the activation of PPARα transcriptional activity [27,28,47,101,103,104].

Thus, the strategy of increasing PPARα expression and/or activation was proposed as a promising therapeutic approach for ASD. Upregulation of PPARα expression or repeated activation of the receptor by administration of an exogenous agonist is accompanied by blunting neuroinflammatory processes and restored redox balance in the PFC and hippocampus, which correlates with improved behavioral deficits, both in environmentally induced or genetic animal models [13,101,103,104]. Moreover, PEA, which effectively reduces inflammatory responses [105] and depressive-like behaviors [43,58], has been successfully tested in animal models of ASD. In the genetic model of the BTBR mouse, PEA administration dose-dependently reduces perseverative and stereotypic responses and improves social behaviors by a PPARα-dependent mechanism [102]. Similar positive effects were reported in the valproic acid (VPA)-induced mouse ASD model [106].

In addition to the effects on neuroinflammation, PEA-mediated activation of PPARα has also been related to an upregulation of BDNF-mediated transmission in the hippocampus of BTBR mice. PEA administration increased mRNA and protein expression of BDNF and TrkB and the phosphorylation levels of CREB [102]. The BDNF-activated signaling pathways may contribute to the positive effects induced by PEA administration via expression upregulation of genes that mediate enhanced synaptic formation and function and pro-survival effects. Similar effects have been demonstrated for the synthetic PPARα ligand fenofibrate that also improved social behaviors by increasing BDNF signaling and phosphorylated CREB expression in the hippocampus in a mouse model of chronic social defeat stress [91]. Moreover, PEA may also ease the behavioral impairments and the neurochemical alterations characteristic of the ASD phenotype through reducing glutamate toxicity and thereby providing neuroprotective effects [28,107]. This bimodal effect on neuroinflammation and glutamate signaling represents an additional neurobiological mechanism that supports PEA’s putative clinical benefit in ASD [108] (Table 2).

The role of PEA following PPARα activation in ASD has been explored in a limited number of clinical studies. A single observational study reported lower serum levels of PEA and other endocannabinoids in ASD children and adolescents compared to controls of similar sex and age [109]. Few interventional studies reported the positive behavioral effects of PEA supplementation as monotherapy or add-on therapy to antipsychotic treatment with an overall reduction in the severity of autism symptoms [106,108,110,111] (Table 2).

The effects of fenofibrate administration in the VPA-induced rat model of ASD have also been examined in a study that challenged the social motivational theory of ASD [84]. The social motivational theory ascribes the core social deficits of the disorder to the severe impairment of the social reward-processing mechanisms that drive sociality [112,113,114]. The pro-motivational activity of fenofibrate was demonstrated in a model of stress-induced motivational anhedonia [72]. In the ASD model, repeated administration with fenofibrate, from weaning to late adolescence, rescued the social deficits and restored the dopaminergic response to social stimuli, impaired by prenatal VPA exposure [84]. These results demonstrate that a treatment able to relieve motivational anhedonia in an animal model of depression also relieves social impairments in a model of ASD. Thus, this study supports the relevance of social anhedonia in the development of the ASD phenotype and suggests a rationale for early pharmacological interventions that target core social symptoms in order to facilitate motivational mechanisms. PPARα levels were decreased in the VTA region after 4-week fenofibrate administration and suggest that a feasible mechanism underlying fenofibrate pro-motivational effects may be the PPARα-mediated regulation of VTA dopaminergic neuron activity, as outlined above (*PPARα regulation of VTA dopamine neurons*).

### 3.3. Role of PPARα in Schizophrenia

Schizophrenia is a heterogeneous clinical syndrome characterized by a range of cognitive, behavioral, and emotional dysfunctions. The etiology of this disorder is multifactorial with a significant genetic burden, resulting from the cumulative effects of hundreds of genes dispersed across the genome, each with small size effects [115] and interacting with several environmental elements. These factors may result in developmentally mediated changes in neuroplasticity that are conducive to dysfunctional circuit maturation and impaired connectivity. Thus, schizophrenia can be regarded as a neurodevelopmental disorder elicited by aversive events in early life, at birth, or during the intrauterine life, and this theory is supported by large birth cohort studies that begun from the 1940s onwards [116,117,118,119]. Several studies documented a crucial role of PPARα during neurodevelopment. Intriguingly, the *PPARA* gene is highly expressed in the mouse brain during the developmental period and reaches a peak at embryonic day 16.5 before declining in early postnatal life [49]. Furthermore, the orthologs of mammalian PPARα regulate the formation of neurons and glial cells in the zebrafish [120]. In vitro studies showed that all the three isoforms of PPAR are expressed in mammalian neural stem cells, and PPARα is involved in neural stem cell proliferation, migration, and differentiation consistent with its role in proliferation, differentiation, and death of different cell types [121]. These results suggest the hypothesis that impairments in PPARα function may contribute to the pathophysiology of schizophrenia as a result of its role in neurodevelopment. In support of this hypothesis, several animal models of PPARα deficiency show endophenotypes that resemble symptoms of schizophrenia in humans. Prepulse inhibition (PPI) is a common measure of sensorimotor gating that is decreased in schizophrenic patients and is often reproduced in experimental models of schizophrenia. PPARα KO mice exhibit a decrease in startle reflex in the PPI test and an increase in perseverative and repetitive behaviors and cognitive deficits indicative of impaired cognitive flexibility [49,101]. Pharmacological stimulation of PPARα rescues the endophenotypes of schizophrenia in several KO mouse models [49,101]. Moreover, PPARα KO mice show abnormalities in dendritic spine maturation [49]. This finding may be related to PPARα involvement in the regulation of synaptogenesis-related genes, including cyclic AMP response element-binding 1 (Creb1) [49]. In addition, PPARα KO mice present reduced expression of Sumo1 (encoding small ubiquitin-related modifier 1), that plays an important role in the maturation of dendritic spines [49].

In the past years, PPARα agonists have been proposed for repurposing as potential agents for the treatment of neuropsychiatric disorders [27,49,122] and neurodegenerative diseases [28], in consideration of their role in the regulation of gene expression in pathways that control oxidative stress and neuroinflammatory responses. Indeed, the development of schizophrenia has also been related to the consequences of early pathological inflammatory processes that induce oxidative stress and reduce cellular antioxidant mechanisms, resulting in permanent cell damage. Dysregulation of the glutathione pathway, reduced levels of antioxidant enzymes, and lipid peroxidation have long been documented in schizophrenic patients [123,124,125,126,127]. Genetic studies have shown associations between allelic variations in genes codifying enzymes involved in glutathione biosynthesis/metabolism and schizophrenia [128,129].

Consistent with these data, neonatal or perinatal oxidative stress injuries in rodents, which model obstetric complications at birth or glutathione deficit, induce a delayed onset schizophrenia-like phenotype, including specific cognitive deficits, such as PPI dysfunctions [130]. In a condition that has been proposed as a neurodevelopmental model of schizophrenia, the PPI deficits induced in rats by kainic acid administration at postnatal day 7 are reduced by long-term fenofibrate treatment [131]. However, no other studies have further addressed the effect of PPARα agonists using this specific model of schizophrenia.

The role of PPARα in the development of schizophrenia is also supported by the positive effects of PPARα agonist administration, in a study conducted in a neurodevelopmental model of this disorder [132]. Maternal immune activation (MIA) was induced in rats by in utero exposure to polyriboinosinic-polyribocytidylic acid (Poly I:C), that elicits the activation of maternal innate immune response resulting in schizophrenia-like phenotypes in rodents [133]. The deficits in PPI of startle reflex and the dysregulation in dopaminergic transmission were prevented in the offspring by repeated prenatal administration with the PPARα agonist, fenofibrate [132]. One hypothesis on the mechanisms that underlie the effects of fenofibrate proposes that repeated PPARα activation could negatively modulate NFkB and activator protein 1 (AP-1) signaling [134], reconducting the role of PPARα mainly to the modulation of pathways that control oxidative stress and neuroinflammatory responses.

In humans, several studies have tested the association between PPARα polymorphisms and schizophrenia, with conflicting results. The analysis of 35 intronic single nucleotide polymorphisms (SNPs) in the *PPARA* gene suggested a potential role for PPARα in the susceptibility to develop the disorder [135]. A recent study in Japanese patients with schizophrenia identified several *PPARA* variants with reduced functionality, which may increase the risk of developing psychotic symptoms [49]. Thus, these mutations identified in schizophrenic patients, although very rare, support a role for dysfunctional PPARα transcriptional activity in the development of the disorder, in the context of a polygenic condition resulting from the small effect size of numerous genes. However, other studies of *PPARA* SNPs or genome-wide association studies (GWAS) analysis reported no association with schizophrenia [136,137,138].

### 3.4. Neurodegenerative Disorders

Alzheimer’s disease (AD), Parkinson’s disease (PD), Huntington’s disease, and amyotrophic lateral sclerosis are the most prevalent neurodegenerative diseases. All these diseases are characterized by a prominent neuroinflammatory component and are associated with the abnormal accumulation of proteins such as β-amyloid (Aβ), microtubule-associated protein tau, α-synuclein (α-syn), huntingtin, and superoxide dismutase 1 (SOD1). The interest for the possible role of PPARα in neurodegenerative diseases stems from the well-known range of effects elicited by PPARα in the nervous system and outside the nervous system. The prominent role of PPARα in lipid metabolism, fatty acid transport, and catabolism, in addition to its anti-inflammatory and neuroprotective effects, stimulated past and current research to focus on investigating the effects of PPARα activation in AD. This is further substantiated by the evidence that increased LDL and total cholesterol levels are associated with increased risk for neurodegenerative diseases, amyloid precursor protein (APP) deposits, and amyloid-β (Aβ) peptide accumulation [139].

One of the pathological hallmarks of AD is the presence of extracellular senile plaques containing the Aβ peptide (particularly Aβ_40/42_ fragments) generated following the proteolytic processing of its precursor, the APP, by the sequential activity of β- and γ-secretase (amyloidogenic pathway). On the contrary, α-secretase activity produces the soluble form of APP, which is not susceptible to aggregation (non-amyloidogenic pathway). Several studies demonstrated that PPARα plays a protective role in APP brain metabolism via various mechanisms [140,141,142,143]. In a model of AD, the APP/PS1 double transgenic mouse expressing a chimeric mouse/human amyloid precursor protein and a mutant human presenilin 1, and development of relevant behavioral and histopathological features observed in AD patients (e.g., Aβ peptide deposition and cognitive impairment), is accompanied by a decrease in the levels of PPARα in the hippocampus [144]. In addition, PPARα activation by fenofibrate administered for 6 months in APP/PS1 transgenic mice reduced the soluble form of APP and amyloid-β 42 (Aβ42) release by reducing levels of β-secretase, which is the rate-limiting step enzyme for Aβ generation [144] in the amyloidogenic pathway [145]. This finding is supported by the recent observation that the administration of the PPARα agonist GW7647 reduced Aβ deposition and led to a general improvement of performance in cognitive tasks in APP/PS1 mice, likely by reducing iron deposit and protecting neurons against oxidative stress and iron-induced lipid peroxidation [146].

In order to overcome the intrinsic limitations of single animal models that can provide relevant information on the neurobiology of the disease but do not exactly mirror the human behavioral and histopathological phenotype, several rodent models have been developed over the years. The 5XFAD mouse model was developed in 2006 by overexpressing the human APP with three familial AD (FAD) mutations and human presenilin-1 with two familial mutations [147]. The 5XFAD mice express age-dependent amyloidosis, neurodegeneration, neuroinflammation, and cognitive deficits, despite sex divergences with human AD (reviewed by [148]). Using 5XFAD mice null for PPARα, an exacerbation of Aβ aggregation and early plaque formation was observed in comparison to traditional 5XFAD mice. Interestingly, the PPARα agonist gemfibrozil increased the soluble APP form and decreased Aβ production, and these effects are likely due to the beneficial role played by PPARα in promoting the non-amyloidogenic pathway. In particular, it has been suggested that PPARα activation facilitates the proteolysis of APP in hippocampal neurons via transcriptional induction of a specific α-secretase [140], as well as decreases microgliosis and astrogliosis in the hippocampus and cortex [149]. More recently, PPARα has been identified as a factor directly regulating autophagy in the nervous system and, potentially, inducing Aβ clearance. Indeed, gemfibrozil administration for 2 months in the APP/PS1 mice mitigates AD-related pathology and attenuates the associated behavioral deficits, by increasing autophagy in microglia and astrocytes [150]. Moreover, administration of gemfibrozil plus retinoic acid to 5XFAD mice induces lysosomial biogenesis and reduces the Aβ plaque load, leading to improved long-term memory and spatial learning [151].

It is noteworthy to point out that polymorphisms in the *PPARA* gene have been associated with increased risk for AD development, yet the clinical relevance of the specific contribution of the identified genetic variants is still debated. For example, the PPARα L162V genotype seems to influence Aβ levels in cerebrospinal fluid [152], however, this was not replicated in another study [153]. Moreover, in the brain of AD patients PPARα expression levels are significantly reduced [154] and show an inverse correlation with APP expression in AD patients, but not in control subjects [155]. These preclinical and clinical data suggest that PPARα plays a role in the development and progression of AD, but the underlying mechanisms are still to be clarified.

The role of PPARα in neurodegenerative disorders is also relevant for Parkinson’s disease (PD), given the anti-inflammatory and neuroprotective effects and the role played by neuroinflammation in dopaminergic cell death, which has been demonstrated in different animal models of PD. The protective effect exerted by PPARα agonists on striatal dopaminergic neurons and motor deficits has been shown by different studies in MPTP rodent models of PD [156,157]. Supporting these results, genetic ablation of PPARα worsened MPTP toxicity, although it did not impair motility, while PEA administration reduced motor dysfunction, attenuated apoptosis, and protected from the negative consequences of microglia activation [46].

In addition, a recent study demonstrated that the positive effects of the PPARα agonist gemfibrozil on MPTP-induced deficits are mediated by increased transcription of the glial cell-line-derived neurotrophic factor (GDNF) gene in astrocytes [158].

Conversely, limited data are available on the involvement of PPARα in other neurodegenerative diseases. In a model of Huntington disease generated by administration of nitro-propionic acid [159], fenofibrate improved the motor deficits and cognitive dysfunction due to reduction in pro-inflammatory molecules such as IL-1β and TNFα [160]. A similar underlying mechanism was likely implicated in a model of amyotrophic lateral sclerosis, where fenofibrate slowed the progression of neurodegeneration by upregulating and downregulating, respectively, neuroprotective (cytokine signaling suppressing, anti-inflammatory, and anti-oxidative genes in the spinal cord) and inflammatory genes (inducible NOS and cyclooxygenase [47].

Overall, these observations suggest that PPARα may be a promising target for the treatment of neurodegenerative diseases characterized by increased inflammatory processes.

## 4. Concluding Remarks

PPARα, a nuclear receptor and a transcription factor involved in the modulation of thousands of genes, has a role in several pathophysiological functions spanning from regulation of inflammatory processes to oxidative stress, and newly emerged as a neuronal target to improve cognitive and emotional behavioral deficits. Several mechanisms have been suggested for the beneficial behavioral effects induced by activation of the PPARα signaling cascade. Following activation by endogenous or synthetic ligands, PPARα exerts a potent anti-inflammatory action. While this is the best characterized action triggered by PPARα, its activation affects several other signaling pathways, including elevation of BDNF expression and stimulation of neurosteroid biosynthesis. Clinical trials to study the efficacy of PPARα agonists in the treatment of mood, neurodevelopmental, and neurodegenerative disorders are emerging and promise to provide evidence to support novel pharmacological strategies for the therapeutic management of conditions that share a strong neuroinflammatory component, including major depression, PTSD, and ASD, but also PD and AD. However, translation of this therapeutic strategy to the clinic may not be straightforward, as animal models do not entirely recapitulate the neurobiology and pathology of neuropsychiatric diseases in all their complexity, and preclinical studies often use as experimental design a preventive rather than a treatment strategy. Thus, a crucial issue in preclinical study design is the stage of the disorder when to begin treatment, considering real-world clinical practice. Another issue to consider is the potential peripheral adverse effects of PPARα agonists, which may require trials dedicated to careful dose-finding and establishing optimal combination therapies with drugs that mitigate the peripheral adverse effects.

## Figures and Tables

**Table 1:** Overview of preclinical animal models and primary findings of studies providing evidence for a role of PPARα in neuropsychiatric and neurodegenerative diseases.

***PPARα Agonist***	***Disease***	***Preclinical Model***	***Output Features***	***Ref.***
**PEA −/+ luteolin**	*Depression*	Corticosterone-induced depression	Increased neurogenesis	[52]
**Fenofibrate**		Chronic unavoidable stress in rat	Regulation of VTA dopamine neurons	[72]
**Fenofibrate**		Chronic social defeat in mice	Normalization of BDNF signaling in hippocampus	[91]
**Gemfibrozil**		Chronic mild stress in mice	Normalization of BDNF signaling in hippocampus	[92]
**PEA**		Social isolation	Increase in steroidogenesis in limbic areas	[48]
**WY-14643** (synthetic ligand)		(LPS)-induced depressive-like behaviors	Prevention of neuroinflammation and oxidative stress	[60]
**Fenofibrate**	*Autism* *Spectrum* *Disorder*	Propionic acid model	Reduction in oxidative stress and neuroinflammation	[103]
**PEA + luteolin**		Valproic acid model	Reduction in inflammation and apoptosis	[106]
**PEA**		BTBR T+tf/J mice	Modulation of neuroprotection, inflammation, and gut–brain axis.	[102]
**Fenofibrate**		Valproic acid model	Reinstatement of dopaminergic response to social stimuli	[84]
**Fenofibrate**	*Schizophrenia*	Kainic acid model	Reduction in behavioral impairment	[131]
**Fenofibrate**		Maternal immune activation	Modulation of pathways underlying oxidative stress and neuroinflammation	[132]
**Fenofibrate**	*Alzheimer Disease*	APP/PS1 transgenic mice	Reduction in Ab deposition and levels of b-secretase	[142]
**GW7647**		APP/PS1 transgenic mice	Reduction in Ab deposition, improved cognition, and decrease in oxidative stress	[146]
**Gemfibrozil**		5XFAD mice	Proteolysis of APP by stimulating a-secretase	[140]
**Gemfibrozil**		5XFAD mice	Reduction in a microgliosis and astrogliosis in hippocampus and cortex	[149]
**Gemfibrozil**		APP-PSEN1DE9	Reduction in Ab accumulation and improved cognitive impairment by modulation of autophagy	[150]
**Gemfibrozil + retinoic acid**		5XFAD mice	Astroglial uptake and degradation of Ab	[151]
**Fenofibrate**	*Parkinson’s Disease*	MPTP	Prevention of MPTP-induced dopaminergic loss in SNpc	[156]
**Fenofibrate**		MPTP	Reduced hypolocomotion, oxidative stress, and degeneration of dopamine neurons in SNpc	[157]
**PEA**		MPTP	Reduction in MPTP-induced microglia activation and motor deficits	[46]
**Gemfibrozil**		MPTP	Neuroprotection via GDNF pathway	[158]

**Table 2:** Overview of primary findings in clinical studies providing evidence for a role of PPARα in neuropsychiatric disorders.

***Drug***	***Disease***	***Clinical Study***	***Output***	***Ref.***
**Ultramicronized PEA + Citalopram**	*Depression*	Randomized, double-blind placebo-controlled trial	As add-on therapy to antidepressant treatment, PEA increased antidepressant response rate	[61]
**Ultramicronized PEA**	*Autism* *Spectrum* *Disorder*	Two cases report	Beneficial effects on expressive language and cognition	[110]
**Ultramicronized PEA + luteolin**		A case report	Improvement of ASD symptoms	[106]
**Ultramicronized PEA + Risperidone**		Randomized, double-blind placebo-controlled trial	As add-on therapy to antipsychotic treatment, PEA reduced autism-related irritability and hyperactivity	[111]

## Data Availability

Not applicable.
